# Statistical significance does not imply a real effect

**DOI:** 10.1007/s40037-016-0256-6

**Published:** 2016-03-16

**Authors:** Jimmie Leppink, Kal Winston, Patricia O’Sullivan

**Affiliations:** 1 Maastricht University, Maastricht, The Netherlands; 2 University of Liverpool Online, Amsterdam, The Netherlands; 3 University of California, San Francisco, USA

The overall purpose of the ‘Statistical Points and Pitfalls’ series is to help readers and researchers alike increase awareness of how to use statistics and why/how we fall into inappropriate choices or interpretations. We hope to help readers understand common misconceptions and give clear guidance on how to avoid common pitfalls by offering simple tips to improve your reporting of quantitative research findings. Each entry discusses a commonly encountered inappropriate practice and alternatives from a pragmatic perspective with no mathematics involved. We encourage readers to share comments on or suggestions for this section on Twitter, using the hashtag: #mededstats.

The statement that a statistically significant outcome in a small sample must reflect a real effect is misleading and may result in overconfidence in findings obtained in small-sample studies. We recommend caution in drawing inferences for educational practice from small samples, and provide some practical tips for reporting at the end of this entry.

We must remember that rejecting a null hypothesis is always accompanied with a chance that the null hypothesis was actually true. In a study, we take a sample to estimate results for the whole population from which the sample is taken. Means, correlation coefficients, and other sample findings tend to be imprecise estimates of the corresponding population parameters of interest when sample sizes are small [1]. As recognized by most researchers, small samples tend to have very limited statistical power for detecting population differences (e.g., between groups) and relations (i.e., between variables) of interest. That is, even if a rather substantial difference or relation exists in the population from which we sample, small samples often fail to obtain a statistically significant difference or relation. Consequently, using small samples, we will often fail to reject the null hypothesis of no difference or no relation when that null hypothesis is not true. This testing error is called a *Type II error* [1], familiar to most researchers. So when a small sample size produces a significant difference, researchers erroneously conclude, ignoring the possibility of Type II error, that the difference must reflect a real effect.

However, researchers appear to be less aware of the fact that of all statistically significant findings obtained, a larger portion results in *Type I errors* (i.e., rejecting a null hypothesis that is true) in the case of small samples when compared with samples of a larger size. It is important to understand this issue, as it challenges the aforementioned misconception that a statistically significant outcome in a small sample must reflect a real effect. Let us, therefore, elaborate on this issue in a bit more detail.

## What is the proportion of Type I error?

The statistical significance level used in educational research is typically 5 % (*α* = 0.05). This means that *if the null hypothesis is true*, we will still obtain a statistically significant result and thus commit a Type I error in about one of every 20 samples regardless of sample size. For a small sample, we will have *the same number of incorrect rejections but a smaller number of correct rejections* (more Type II errors) when compared with large samples. Thus, even though we start with the same Type I error rate before collecting any data (here: 5 %, meaning one in every 20 true null hypotheses will be rejected), the expected proportion of Type I errors in a pile of rejected null hypotheses (i.e., statistically significant results) is always larger when using small samples than when using larger samples. Let us demonstrate this with the numerical example summarized in Table 1.

Suppose that two researchers, A and B, decide to each conduct an experiment to compare two types of objective structured clinical examinations (OSCEs) in terms of how much learning they evoke. Researcher A runs the experiment with 64 residents per condition, whereas researcher B has only 11 residents per condition. If there is a difference between the two OSCEs in the population and we draw many, many samples of the same size from that population, statistical power is the proportion of samples that yields a statistically significant outcome and thus (correctly) calls for the null hypothesis to be rejected. In the case of a so-called ‘medium size’ or half a standard deviation difference at population level, software such as G*power [2] will tell you that a traditional two-sided *t*-test with a statistical significance level *α* = 0.05 yields a statistical power of 0.80 for researcher A and a statistical power of 0.20 for researcher B. In other words, if we were to run researcher A’s study many, many times, we would expect to find a statistically significant result in 80 % of the cases, while running researcher B’s study many, many times would result in a statistically significant result in only 20 % of the cases.

Note that these numbers of 80 % and 20 % take as starting point that the null hypothesis is not true, which is the case when at population level there is half a standard deviation difference and the null hypothesis states that there is no difference. However, we usually do not know beforehand whether the null hypothesis is true or not. For ease of calculation, suppose that we test 100 null hypotheses of which 20 are true and 80 are not true because in the latter cases there is half a standard deviation difference between conditions under comparison where the null hypothesis states there is no difference. For all tests, we use the conventional statistical significance level of *α* = 0.05.

**Table 1:** Scenario A (*n* = 64 per condition) and scenario B (*n* = 11 per condition) in terms of expected proportions of Type I error prevalence in a pile of statistically significant outcomes.

Scenario	A (*n* = 64)	B (*n* = 11)
Expected rejections of the 20 true null hypotheses	1	1
Expected rejections of the 80 untrue null hypotheses	64	16
Expected proportion of Type I errors in the pile of statistically significant results	1/65 (≈ 0.015)	1/17 (≈ 0.059)

Using researcher A’s scenario (*n* = 64 per condition) as starting point, we expect to reject one of the 20 true null hypotheses (*α*= 0.05; 1 = 0.05 × 20) and 64 of the 80 untrue null hypotheses (power of 0.80; 64 = 0.8 × 80). Using researcher B’s scenario (*n* = 11 per condition), we also expect to reject one of the 20 true null hypotheses (*α*= 0.05; 1 = 0.05 × 20) but only 16 of the 80 untrue null hypotheses (power of 0.20; 16 = 0.2 × 80). Table 1 summarizes the comparison.

Thus, scenario A is expected to result in a pile of 65 statistically significant findings, one of which is a Type I error, whereas in scenario B we expect a pile of 17 statistically significant findings, one of which is a Type I error. The rates of 1/65 and 1/17 will be different when assuming a different ratio of true/untrue null hypotheses and depends on the sample sizes of the two scenarios as well as on the statistical significance level one chooses. Apart from the illogical scenario in which there are no true or no untrue null hypotheses, the rate of Type I errors in a pile of statistically significant findings is always expected to be higher in small samples than in large samples.

## To conclude

We should always be wary of interpreting a statistically significant effect as reflecting a real effect but even more so in the case of small samples. Of course, logistic factors (e.g., time constraints, only a limited number of subjects available) sometimes limit options in terms of sample size (e.g., two groups of ten subjects or a linear correlation in a single sample of 15 subjects). However, whatever the reason for using small samples, we recommend caution in drawing inferences for educational practice. Reporting confidence intervals may help both authors and readers to appreciate the uncertainty around sample estimates, especially for small samples [3]. Reporting estimates of effect size (i.e., the strength of a relation or effect of interest expressed in statistical units such as standard deviations) may help to do power analyses and required sample size analyses for subsequent studies, but they are not immune from the effects of poor parameter estimates arising from small samples [4]. Replication studies and meta-analyses [5] are commonly good options to consider—a series of studies generally provides more accurate estimates than a single study—but even more so when dealing with small samples.
